# Application of feature selection methods for automated clustering analysis: a review on synthetic datasets

**DOI:** 10.1007/s00521-017-3005-9

**Published:** 2017-04-22

**Authors:** Aliyu Usman Ahmad, Andrew Starkey

**Affiliations:** School of Engineering, University of Aberdeen, Aberdeen, UK

**Keywords:** Clustering, Self-organising neural network map, Feature selection, Automation

## Abstract

The effective modelling of high-dimensional data with hundreds to thousands of features remains a challenging task in the field of machine learning. This process is a manually intensive task and requires skilled data scientists to apply exploratory data analysis techniques and statistical methods in pre-processing datasets for meaningful analysis with machine learning methods. However, the massive growth of data has brought about the need for fully automated data analysis methods. One of the key challenges is the accurate selection of a set of relevant features, which can be buried in high-dimensional data along with irrelevant noisy features, by choosing a subset of the complete set of input features that predicts the output with higher accuracy comparable to the performance of the complete input set. Kohonen’s self-organising neural network map has been utilised in various ways for this task, such as with the weighted self-organising map (WSOM) approach and this method is reviewed for its efficacy. The study demonstrates that the WSOM approach can result in different results on different runs on a given dataset due to the inappropriate use of the steepest descent optimisation method to minimise the weighted SOM’s cost function. An alternative feature weighting approach based on analysis of the SOM after training is presented; the proposed approach allows the SOM to converge before analysing the input relevance, unlike the WSOM that aims to apply weighting to the inputs during the training which distorts the SOM’s cost function, resulting in multiple local minimums meaning the SOM does not consistently converge to the same state. We demonstrate the superiority of the proposed method over the WSOM and a standard SOM in feature selection with improved clustering analysis.

## Introduction

Clustering is one of the most widely used data analysis methods for numerous practical applications in emerging areas [1]. Clustering entails the process of organising objects into natural groups by finding the class of objects such that the objects in a class are similar to one another and dissimilar from the objects in another class [2]. A clustering algorithm usually considers all input parameters in an attempt to learn as much as possible about the given objects.

The self-organising neural network map (SOM) by Kohonen [3] has been widely used as one of the most successful clustering methods with strong data exploration and visualisation capabilities [4]. The SOM’s mapping preserves a topological relation by maintaining neighbourhood relations such that patterns that are close in the input space are mapped to neurons that are close in the output space and vice-versa.

One of the biggest drawbacks of the SOM algorithm is its inability to automatically identify the features that are relevant for analysis and discard the irrelevant inputs that negatively distort the analysis result [5]. In an attempt to resolve this, researchers [6–8] have worked on the improvement of the algorithm by a feature weighting method during training with the application of the steepest descent optimisation method for the identification of important inputs for clustering (WSOM, weighted self-organising map). The core of the weighted method lies in attempting to describe the contribution of each feature in the clustering algorithm in order to improve the clustering result.

This paper investigates the application of an existing weighted method approach (WSOM) and proposes an alternative approach for identifying the key features in a number of artificially produced datasets and the real world dataset used in the original WSOM study [6–8]. The study demonstrates how information on what the learning algorithm has learnt can be used to identify what is important for the learning, and therefore applied to improve the algorithm’s ability to correctly classify and identify patterns in the data.

## Neural network clustering methods

### Self-organising neural network map

The self-organising neural network map (SOM) is an unsupervised artificial neural network learning method trained to produce a low-dimensional representation of high-dimensional input samples [4].

**Fig. 1:**
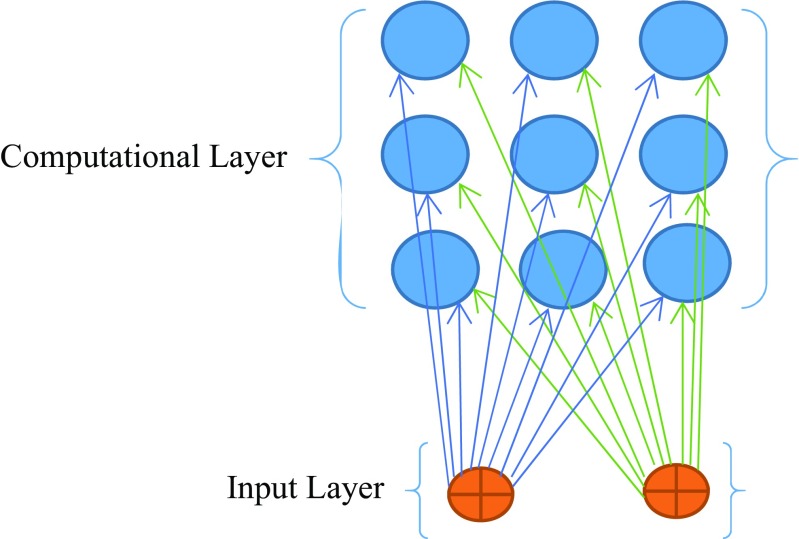
A 2-dimensional self-organising map architecture

A typical SOM consists of the computational layer (map) and the input layers as shown in Fig. 1.

The input layer comprises of the source nodes representing the sample’s features/attributes. There are as many weights for each node as there are number of features (dimensions) in the input layer, represented in the form of an input vector, i.e. *x* = [*x*_*i*_^1^,  *x*_*i*_^2^,  … , *x*_*i*_^*d*^] for an input sample where *d* denotes sample dimensions and *i* the sample number and *n* denoting the total number of samples.

The computational layer (map) consists of neurons placed in nodes of a 2-dimensional grid (lattice); each neuron is identified by its index position, i.e. *j*, on the map and associated with a weight vector, i.e. *W*_*j*_ = {*w*_*ji*_ : *j* = 1,  … , *n*; *i* = 1,  … , *d*} , the size of which is equal to the dimension of the input vector. The set of weights *W* parameters are determined by iteratively minimising the cost function below;

1 $$ R\left( C, W\right)=\sum_{i=1}^N\sum_{j=1}^{\left| W\right|}{\kappa}_{j, c\left({x}_i\right)}{\left\Vert {x}_i-{w}_j\right\Vert}^2 $$

At every *n*th training step, the Gaussian neighbourhood function is calculated for the map; this is expressed as:

2 $$ {K}_{j,\mathrm{c}\left({x}_i\right)\kern0.5em (n)}=\alpha (n)\cdot \mathrm{e}\left(-\frac{{\delta^2}_{j,\mathrm{c}\ \left({x}_i\right)}}{2\sigma {(n)}^2}\right) $$

Where

- ${K}_{j,\mathrm{c}\ \left({x}_i\right)\ (n)}$ is the neighbourhood function between each unit (*j*) on the map and the winning unit c (*x*_*i*_) at the *n*th training step
- ${\delta}_{j,\mathrm{c}\ \left({x}_i\right)}$ is the distance (Euclidean) from the position of unit (*j*) to the winning unit c (*x*_*i*_) on the map.
- *σ*(*n*) is the effective width of the topological neighbourhood at the *n*th training step; this serves as the moderator of the learning step during training iterations. The size of the effective width shrinks with time to facilitate the convergence of the map.
- *α*(*n*) is the learning rate that depends on the number of iterations (*n*); this is initialised to a value of around 0.1 which decreases from *α*_max_ to *α*_min_.

It is possible to use the results of a trained SOM in order to estimate the relevance of feature variables (weights). This is achieved by the use of the quantization error method [9] which is used to analyse the final result of the standard SOM for the identification of the features that were relevant during training.

## Related work

Irrelevant input features are one of the major factors that distort the ability of learning algorithms for pattern recognition in data, as investigated by [10–13]. Numerous researchers have proposed feature selection methods for identifying and selecting the most important inputs in a data that best maximises the performance of learning algorithms [14–16] and can be categorised into filter, wrapper and embedded methods.

Filter methods aim to use statistical approaches to identify what inputs are important and are independent of the classifier, usually applied in the data pre-processing stage prior to training. Some of the commonly used filter methods include the RELIEF algorithm [17], correlation-based feature selection (CFS) [18], fast correlated-based filter (FCBF) [19] and the INTERACT [20] methods. These are further discussed and compared by [21]. Other filter methods include mutual information [22] and Pearson’s correlation coefficient scores [23]. Recently, hybrid methods are introduced; these combine the filter methods with a wrapper method for feature selection; some examples of such methods are presented by [24, 25].

Wrapper methods unlike filter methods aim to identify important inputs by searching for the best subset of features that produce the highest model classification accuracy. Commonly used wrapper methods include the recursive feature elimination method (RFE) [26] and exhaustive search and greedy forward search methods discussed in [27, 28]. Improvements to the above-discussed methods are reviewed and discussed by [29]. Recently emerged wrapper methods include polygon-based cross-validation (CV) with SVM method [30] and competitive swarm optimizer (CSO) [31].

The embedded methods learn about the importance of inputs from the model’s training process and one of the examples of this method is the weighted self-organising map (WSOM) [8] that is investigated in this study. Other methods include hold out SVM (HO-SVM) and kernel-penalized SVM (KP-SVM) reviewed and discussed in [32].

## Methodology

Most of the above methods require manual experimentation that consumes the bulk of the effort invested in the entire clustering analysis, with the exception of the embedded methods that identify important inputs from what the model has learnt. These latter methods are the area of focus in this study which aims to achieve the goal of a fully automated clustering process. We are particularly interested in the use of the self-organising map for automated feature selection due to its powerful topology preservation property, with the neighbourhood function (Eq. 2) that enables the SOM to not only group the data but also illustrates the underlying structure of the data for visualisation. As discussed in [33], the SOM has been widely applied especially for complex and high-dimensional datasets where traditional clustering methods are insufficient.

### SOM weights analysis with quantization error method

On completion of SOM training which is achieved using the batch training method [3], the node weights values are expected to be the representation of their matching input samples, and also relatively close to the input samples mapped to their neighbouring nodes and relatively far from the input samples mapped to distant nodes.

Let *M*_*j*_ be set of the training samples *x*_*i* _ mapped to node *j*, and the quantization error for node *j* is calculated after SOM training as follows:

3 $$ {E}_j={\sum_{M_j}\left\Vert {x}_i-{w}_j\right\Vert}^2 $$

The weight features with the lowest quantization error are expected to be the features whose corresponding input sample features are most relevant when comparing the samples against their winning nodes. A further analysis was carried out on the quantization error values for all the node weights in order to automatically separate the group of the relevant inputs from the irrelevant inputs, a parametric statistical test with median split was carried out on the quantization error values to differentiate the group of high values (as irrelevant features) from the group of low values (as relevant features). Since there is no reliance on a hard-coded threshold value to determine irrelevant and relevant features, this means that this step could be used for any amount of data features and results in a fully automated step for this aspect of the process.

### Weighted self-organising neural network map

The weighted SOM (WSOM) function proposed by [8] is another method designed to compute the relevance of feature variables (weights) automatically during the training process. This approach entails the use of additional random weights that are multiplied by the input weights as a metric for measuring the relevance of the observations during training, and since the comparison is done one sample at a time, the updating method for the WSOM is incremental rather than batch as in the standard SOM.

Let ${\mathfrak{R}}^{\mathrm{d}}$ be the Euclidean data space and *Ε* = { *x*_*i*_; *i* = 1,  … , *Ν*} a set of observations, where each observation *x*_*i*_ = (*x*_*i*_^1^,  *x*_*i*_^2^,  … , *x*_*i*_^*d*^) is a vector in ${\ \mathfrak{R}}^{\mathrm{d}}$.

Each node *j* has prototype weights  *w*_*j*_ = (*w*_*j*_^1^,  *w*_*j*_^2^,  … , *w*_*j*_^*d*^), and a single random weight is assigned to for each input attribute such that; *π*_*d*_ = (*π*_1_, *π*_2_, …*π*_*d*_).

This method attempts to find the relevance of all the weights of a single vector which are applied against the whole set of input weights but is not able to determine the relevance of an individual weight of each node *j* in a trained SOM.

The set of weights *W* and *π* parameters are determined by iteratively minimising the cost function as follows:

4 $$ {R}_{gvw}\left( C, W,\pi \right)=\sum_{i=1}^{\left| E\right|}\sum_{j=1}^{\left| W\right|}{\kappa}_{j, c\left({x}_i\right)}{\left\Vert {\pi}_d\otimes {x}_i-{w}_j\right\Vert}^2 $$

The cost function *R*_*gvw*_ ( *W*, *π*) is as described in Eq . 4. The algorithm is optimised by finding the $$ \begin{array}{c} \min \\ {}\  W,\pi \end{array}\ {\  R}_{gvw}\ \left(\  W,\pi \right) $$. The process begins by initially starting with some random values for *W* , *π* then these values are modified in order to reduce *R*_*gvw*_ ( *W*, *π*), until the minimum of the cost function is reached.

The method uses the steepest descent algorithm in order to optimise its cost function;

{

5 $$ {R}_j:={R}_j-\alpha \frac{\partial }{\partial {R}_j}{R}_{gvw}\left( W,\pi \right)\kern1em \left(\mathrm{For}\kern0.5em  j= W\kern0.5em \mathrm{and}\kern0.5em  j=\pi \right) $$

}

The gradient descent minimization of the function can be implemented as;

6 $$ {w}_j\left( n+1\right):\kern0.5em ={w}_j(n)-(n)-\alpha (n){\kappa}_{j, c\left({x}_i\right)}(n){\kappa}_{j, c\left({x}_i\right)}\left({w}_j-{\pi}_g\otimes {x}_i\right) $$

7 $$ {\pi}_g\left( n+1\right):\kern0.5em ={\pi}_g(n)-(n)-\alpha (n){\kappa}_{j, c\left({x}_i\right)}(n){\kappa}_{j, c\left({x}_i\right)}\kern0.5em {x}_i\left({\pi}_g\otimes {x}_i-{w}_j\right) $$

The steepest descent algorithm which is utilised by the WSOM method searches for the minimum of a function by computing the gradient of the function, starting at a random point *P*_0_, and moving from *P*_*i*_ to *P*_*i* + 1_ in the direction of the local downhill gradient −∇*f*(*P*_*i*_) for each iteration of line minimization.

**Fig. 2:**
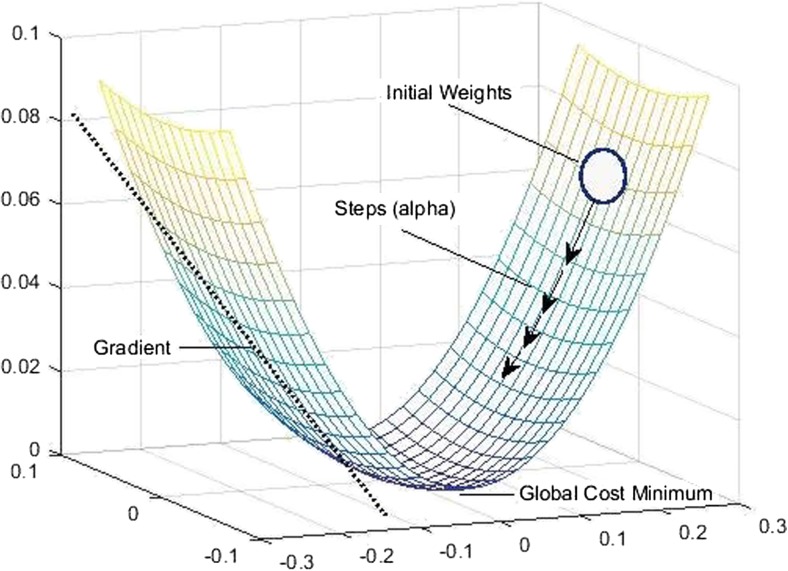
Steepest decent method for a quadratic functions

**Fig. 3:**
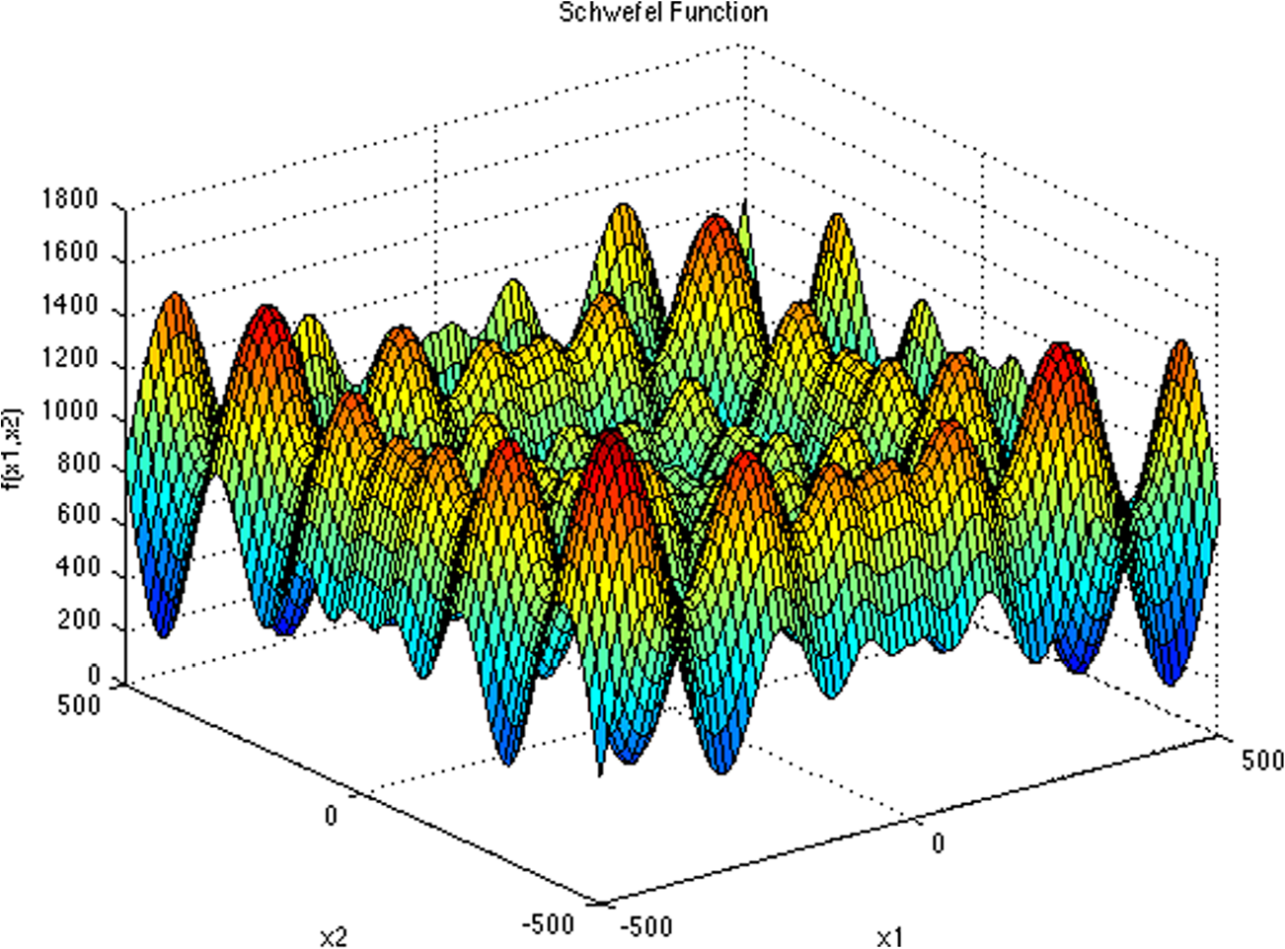
Problems beyond quadratic functions, Schwefel function [35]

The steepest descent method is guaranteed to find a solution for quadratic functions, which are convex-shaped functions with a single minimum that is equal to the global minimum [34] (as illustrated in Fig. 2). For problems beyond quadratic functions with multiple local minimums (such as Schwefel function, Fig. 3), the gradient descent finds the solution of a function based on the first identified local minimum and ignores other local minimums, and does not necessarily and cannot be guaranteed to find the global minimum of the given function. It is therefore important to confirm that the cost function for the WSOM method results in a single global minimum that can be found by the steepest descent approach.

For a full description of the WSOM process, the reader is directed to [7, 8]. In the WSOM approach, the relevance of an input vector is indicated by the global weights with irrelevant vectors having global weights close to 0 and relevant vectors having global weights different from 0. The relevance of an input vector can be measured by this method only if the given data features are normalised to the same scale.

## Experiment

### Synthetic datasets

In order to assess the efficacy of the WSOM method against a standard SOM implementation, a number of synthetic datasets were developed which had different features, starting with a simple dataset with a small number of attributes and moving to datasets with a larger number of inputs and additional noise. All data sets had equal class distribution (i.e. same number of samples for each class), and normalised, with the exception of Synthetic_Data04 and Synthetic_Data05. These datasets are discussed more fully in the tables. The use of synthetic data rather than real data sets of this type is very important as it allows a full assessment of how well the techniques work and what type of problems they can solve, and where they may encounter difficulties if any.

In addition, the dataset used in the original WSOM paper was obtained which is a real world dataset of waveform data and is also described in Table 1.

**Table 1:** Synthetic and real datasets definition

Dataset name	Samples	Input features	Classes
Synthetic_Data01 (Normalised)	100	4	5
	All classes defined by first 4 related features.		
	This is a simple dataset with no irrelevant inputs and outliers, created mainly for exploring the cost functions of the two self-organising algorithms.		
Synthetic_Data02 (Normalised)	1220	7	5
	All classes defined by first 4 related features. Irrelevant features: 5, 6, 7		
	Irrelevant inputs are clearly separated from the relevant inputs for easy identification by the algorithms.		
Synthetic_Data03 (Normalised)	1220	10	5
	Classes defined by features independently with equal distribution. Class1 = 1, 2, & 3, Class2 = 4, 5, & 6, Class3 = 2, 3, 4 & 5, Class4 = 6, 7, & 8, Class5 = 1, 4, & 8. Noise features; features 9 & 10		
	In addition to Synthetic_Data02, the definition of classes was distributed among variables, to identify the self-organising method’s ability to identify the degree of relevance of the input features for classification.		
Synthetic_Data04 (Normalised)	1220	9	5
	Classes defined by features independently with unequal distribution. Class1 (550 samples) = 1,2, & 3, Class2 (300 samples) = 1, 2, & 3, Class3 (200 samples) = 2, 4, & 5, Class4 (100 samples) = 1, 3, 5, & 6, Class5 (70 samples) = 1,3,4, & 7, Noise features; features 8 & 9		
Synthetic_Data05 (Unnormalised)	1220	7	5
	All classes defined by first 4 related features. Irrelevant features: 5, 6, 7		
	This dataset was created to evaluate the self-organising system’s performance in identifying irrelevant inputs from unnormalised datasets having features of unequal variance.		
WaveForm dataset (Normalised)	5000	40	3
	As described by [36] the first 21 inputs of the waveform data describe the classes, the latter 19 are completely irrelevant noise features with mean 0 and variance 1. More details can be found from the UCI repository online. No information is provided on which inputs out of the first 21 describe each class.		

### Experiment design

As both methods rely on a random process, the performance of the algorithms was measured based on results of 10 runs for each of the methods on the synthetic datasets, and the results of these runs are shown in Tables 2, 3, 4, 5, 6 and 7.

**Table 2:** Performance of clustering methods on Synthetic_Data01

Clustering Synthetic_Data01						
Training parameters	Map dimension, 3 × 3 rectangular grid topology Training epochs, 1000 Learning rate, 0.1					
	Weighted SOM			Standard SOM		
RUNS	Identified important inputs	Correct classes found (all inputs)	Correct classes found (selected inputs)	Identified important attributes	Correct classes found (all inputs)	Correct classes found (selected inputs)
Run 1	1/4	1/5	0/5	4/4	5/5	5/5
Run 2	1/4	1/5	0/5	4/4	5/5	4/5
Run 3	1/4	1/5	1/5	4/4	5/5	5/5
Run 4	1/4	2/5	1/5	4/4	5/5	5/5
Run 5	2/4	1/5	2/5	4/4	4/5	5/5
Run 6	1/4	0/5	0/5	4/4	5/5	5/5
Run 7	1/4	1/5	0/5	4/4	5/5	5/5
Run 8	1/4	0/5	1/5	4/4	5/5	5/5
Run 9	1/4	2/5	1/5	4/4	5/5	5/5
Run 10	1/4	0/5	0/5	4/4	4/5	5/5

**Table 3:** Performance of clustering methods on Synthetic_Data02

Clustering Synthetic_Data02						
Training parameters	Map dimension, 3 × 3 rectangular grid topology Training epochs, 1000 Learning rate, 0.1					
	Weighted SOM			Standard SOM		
RUNS	Identified important inputs	Correct classes found (all inputs)	Correct classes found (selected inputs)	Identified important attributes	Correct classes found (all inputs)	Correct classes found (selected inputs)
Run 1	1/4	0/5	0/5	4/4	2/5	5/5
Run 2	0/4	0/5	–	4/4	1/5	5/5
Run 3	2/4	1/5	2/5	4/4	2/5	5/5
Run 4	1/4	0/5	1/5	3/4	1/5	4/5
Run 5	2/4	0/5	1/5	4/4	1/5	5/5
Run 6	3/4	1/5	1/5	4/4	2/5	5/5
Run 7	1/4	1/5	1/5	4/4	3/5	5/5
Run 8	2/4	0/5	2/5	4/4	2/5	5/5
Run 9	0/4	0/5	–	4/4	3/5	5/5
Run 10	1/4	0/5	1/5	4/4	2/5	5/5

**Table 4:** Performance of clustering methods on Synthetic_Data03

Clustering Synthetic_Data03						
Training parameters	Map dimension, 3 × 3 rectangular grid topology Training epochs, 1000 Learning rate, 0.1					
	Weighted SOM			Standard SOM		
RUNS	Identified important inputs	Correct classes found (all inputs)	Correct classes found (selected inputs)	Identified important attributes	Correct classes found (all inputs)	Correct classes found (selected inputs)
Run 1	0/8	0/5	–	2/8	3/5	2/5
Run 2	0/8	0/5	–	4/8	1/5	3/5
Run 3	1/8	1/5	0/5	2/8	1/5	2/5
Run 4	0/8	0/5	–	3/8	0/5	2/5
Run 5	1/8	0/5	0/5	1/8	1/5	1/5
Run 6	2/8	0/5	1/5	1/8	0/5	1/5
Run 7	1/8	1/5	1/5	5/8	1/5	4/5
Run 8	1/8	0/5	0/5	1/8	2/5	2/5
Run 9	0/8	0/5	–	2/8	1/5	2/5
Run 10	1/8	0/5	0/5	2/8	1/5	2/5

**Table 5:** Performance of clustering methods on Synthetic_Data04

Clustering Synthetic_Data04						
Training parameters	Map dimension, 3 × 3 rectangular grid topology Training epochs, 1000 Learning rate, 0.1					
	Weighted SOM			Standard SOM		
RUNS	Identified important inputs	Correct classes found (all inputs)	Correct classes found (selected inputs)	Identified important attributes	Correct classes found (all inputs)	Correct classes found (selected inputs)
Run 1	2/7	1/5	1/5	4/7	3/5	2/5
Run 2	1/7	1/5	0/5	4/7	2/5	3/5
Run 3	1/7	1/5	0/5	2/7	1/5	1/5
Run 4	1/7	1/5	0/5	4/7	4/5	2/5
Run 5	1/7	1/5	0/5	3/7	2/5	2/5
Run 6	0/7	0/5	–	4/7	2/5	3/5
Run 7	2/7	1/5	1/5	4/7	2/5	3/5
Run 8	1/7	1/5	0/5	2/7	1/5	1/5
Run 9	1/7	1/5	0/5	2/7	1/5	1/5
Run 10	1/7	1/5	0/5	4/7	2/5	3/5

**Table 6:** Performance of clustering methods on Synthetic_Data05

Clustering Synthetic_Data05						
Training parameters	Map dimension, 3 × 3 rectangular grid topology Training epochs, 1000 Learning rate, 0.1					
	Weighted SOM			Standard SOM		
RUNS	Identified important inputs	Correct classes found (all inputs)	Correct classes found (selected inputs)	Identified important attributes	Correct classes found (all inputs)	Correct classes found (selected inputs)
Run 1	2/4	0/5	1/5	3/4	3/5	5/5
Run 2	0/4	0/5	–	4/4	2/5	5/5
Run 3	1/4	1/5	2/5	4/4	2/5	5/5
Run 4	1/4	0/5	1/5	4/4	2/5	5/5
Run 5	1/4	0/5	2/5	4/4	2/5	5/5
Run 6	0/4	1/5	–	4/4	2/5	5/5
Run 7	2/4	1/5	0/5	4/4	2/5	5/5
Run 8	1/4	1/5	1/5	4/4	2/5	5/5
Run 9	0/4	1/5	–	4/4	3/5	5/5
Run 10	1/4	1/5	0/5	3/4	2/5	5/5

**Table 7:** Performance of clustering methods for waveform data

Clustering waveform data					
Training parameters	Map dimension, 26 × 14 rectangular grid topology Training epochs, 1000 Learning rate, 0.1				
	Weighted SOM		Standard SOM		
RUNS	Identified important inputs	Correct classes found (selected inputs)	Identified important attributes	Correct classes found (all inputs)	Correct classes found (selected inputs)
Run 1	2/20	1/3	18/20	1/3	2/3
Run 2	9/20	1/3	18/20	1/3	3/3
Run 3	19/20	2/3	15/20	2/3	2/3
Run 4	11/20	1/3	18/20	1/3	3/3
Run 5	5/20	1/3	18/20	1/3	3/3
Run 6	19/20	2/3	18/20	2/3	2/3
Run 7	15/20	2/3	19/20	1/3	3/3
Run 8	2/20	1/3	18/20	1/3	2/3
Run 9	16/20	2/3	17/20	1/3	2/3
Run 10	11/20	1/3	18/20	1/3	3/3

However, in order to check whether the weighted SOM cost function (Eq. 4) for Synthetic_Data01 is a quadratic function, to be suitable for the steepest descent optimization approach, the cost function was optimised with the simulated annealing algorithm on Synthetic_Data01; a stochastic search method that aims to expose all possible minimums of the function by random search in space, with initial temperature (*T*_o_) = 10.0 , cooling rate (*α*) = 0.99 and maximum iteration (Maxtime) = 1000.

If a cost function has a single global minimum, the best combination of weight values for different runs of the algorithm will be expected to be within the same region and to have a positive linear correlation when compared against each other. Otherwise, if the cost function has multiple local minimums, then the best combination of weight values would be in different regions for different runs of the algorithm and will not be correlated.

The normalised correlation matrix of the final weights from the simulated annealing algorithm was computed to show the similarity of the weights produced from six runs of the algorithm against each other; the same experiment was carried out on the standard SOM cost function for comparison.

When undertaking the correlation analysis for the WSOM method, the raw SOM weights cannot be used directly but must be divided by the corresponding global weights *π*. This step is required since the global weights and WSOM node weights are linked as described in the cost function, and it is possible due to the random nature of the process that different values could be arrived at for these variables whilst still mapping against similar input samples and the node weights could therefore be different from one run to the next.

In addition, the problem with direct comparison of weights at the same index for the six different simulated annealing runs is that nodes are not necessarily localised to a specific index. In a single run, a node might appear in the first index, whilst in a separate run, the same node might appear in a different index. As such, direct comparison of the weights infers the comparison of random unrelated nodes, which are most likely to be not correlated at all times.

To overcome this problem, the indexes of all the nodes weights was re-arranged to correspond to the best matching positions for all the nodes from the six different simulated annealing runs before carrying out the correlation test on the weights.

Let *E* be set of weight values for a given SOM run (${w}_n^i; n=1,\dots N$), were *N* is the total number of weights and *W* is set of weight values for other SOM runs to total a number of SOM runs *R*. The index position *I* of a node in a given SOM when compared against the SOM with weight values *E* is computed as Eq. 8. For completeness, this was executed for all the SOM runs.

8 $$ I=\sum_{n=1}^{\left| E\right|}\sum_{m=1}^{\left| W\dots R\right|}\mathit{\min}\kern0.5em {\left\Vert {w}_n^i-{w}_m^j\right\Vert}^2 $$

A null hypothesis test *Ho* : *ρ* = 0 was conducted with 0.5 significance level to investigate the relationship between the final node weights (i.e. to see if they are correlated or not). Nodes are correlated if their correlation coefficient is different from zero, and therefore means there is linear relationship between the nodes. There is no correlation for nodes with correlation coefficients close to zero.

## Results

**Fig. 4:**
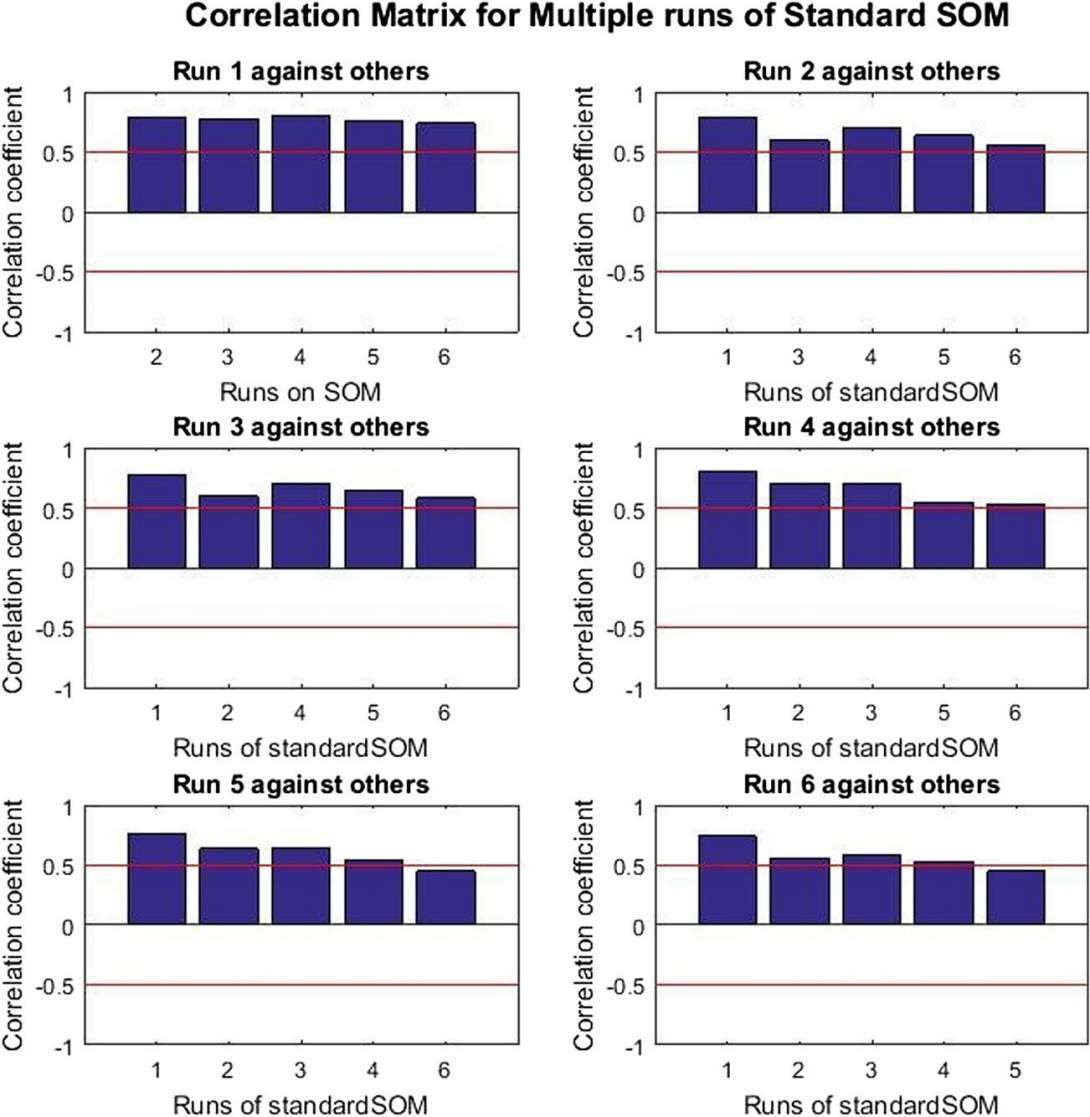
Correlation matrix for multiple runs of standard SOM on Synthetic_Data01

**Fig. 5:**
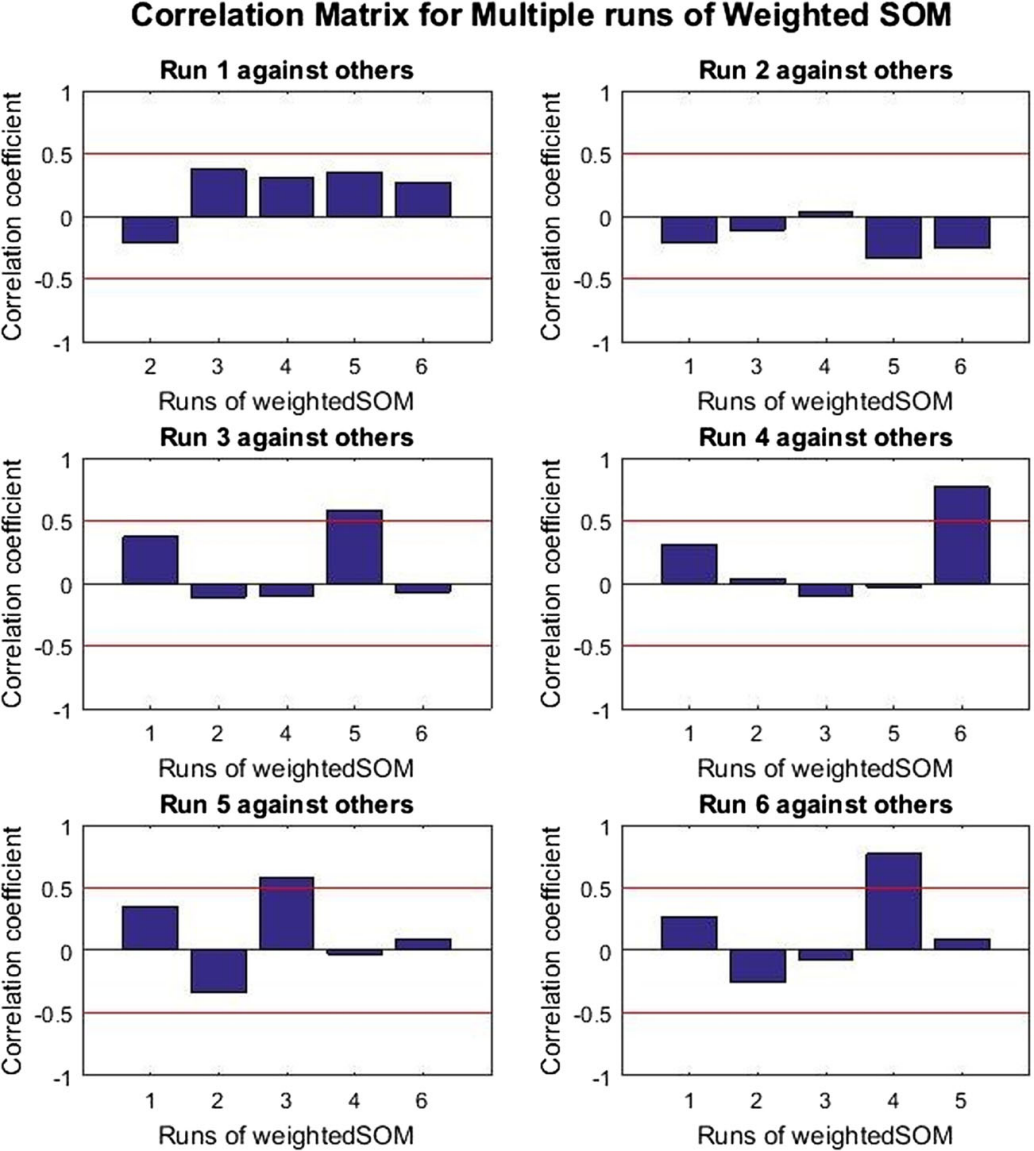
Correlation matrix for multiple runs of weighted SOM on Synthetic_Data01

The analysis of the correlation results can be seen in Figs. 4 and 5 where the bars in the plot represent the correlation coefficients values *ρ* for a given run of the self-organising process compared against another on Synthetic_Data01. The red line on the plots at 0.5 and −0.5 indicate the respective boundaries for positive and negative correlations, with no correlation shown at or around 0.

The bars above the red line indicate the pairs of node weights with correlations significantly different from 0, which implies that there is a significant linear relationship between the weights of the various runs (i.e. the SOM weights are broadly equivalent and the values can be said to be the same). On the other hand, the bars below the line indicate the pairs of node weights with a correlation coefficient that is not significantly different from 0, which implies that there is no significant linear relationship between the weights (i.e. the SOM weights are not the same and contain different values).

The correlation matrix for the standard SOM (Fig. 4) weights shows that almost all pairs of weights (4 out of 6) have correlations significantly different from zero which proves positive correlation among weights. On the other hand, the correlation matrix for the weighted SOM (Fig. 5) shows that only two out of the six weights are correlated, which indicates that this method has resulted in different solutions being found.

In summary, it can be concluded that the simulated annealing algorithm with the standard SOM cost function finds similar solutions in the majority of the different runs. On the other hand, the algorithm with the weighted SOM cost function finds different solutions for most of the runs, which is most likely to be as a result of multiple local minimums in the cost function.

The results given in Tables 2, 3, 4, 5 and 6 for the five synthetic datasets and the real world waveform dataset show the performance of the two methods in identifying the important attributes and whether classes were correctly classified. The tables also give details of the training parameters used. On completion of the classification after training with all inputs, the inputs identified by the respective feature selection method was applied to the dataset so that input samples were remapped to the trained weights with only the selected inputs. If the features have been identified correctly, then it is assumed that the classification of the samples would remain the same. The remapping of input samples was achieved by only recalculating the best matching units (BMUs) [3] for the selected features against their corresponding node weights. Classes are identified if at least 60% of class samples from the input samples belonging to the same class are mapped to the same node.

## Discussion and conclusions

As seen in Table 2, the standard SOM was able to correctly identify all the classes in most of the runs for simple data with no irrelevant inputs and was also able to identify all inputs as important due to low quantization error between weights to their mapped input samples. Unlike the standard SOM, the weighted SOM failed to identify the classes for the same simple data set with no irrelevant inputs. The weighted SOM method also performed poorly by failing to correctly identify clusters and differentiate the relevant input vectors from the irrelevant input vectors on the Synthetic_Data02. However, the standard SOM with quantization error method after training clearly identified the relevant vectors for the training on this dataset. Both methods performed poorly in correctly identifying the clusters in the data as the result of the influence of the irrelevant inputs during the training.

For the more complicated dataset with overlapping class definition (Synthetic_Data03 and Synthetic_Data04), the analysis of the standard SOM’s training result with the quantization error also failed to identify what was important for the training, as presented in Tables 4 and 5.

As discussed in Section 4.2, the steepest descent algorithm is guaranteed to find the local minimum for quadratic functions with a single global minimum, whereas for functions with multiple local minimums, the gradient descent finds the solution of the function based on the first identified local minimum ignoring other local minimums, and therefore is not suitable for the proposed WSOM cost function as our results clearly demonstrate that multiple minimums exist in the solution space defined by the WSOM cost function.

As seen from the performance of methods on Synthetic_Data03 and Synthetic_Data04, identifying feature relevance based on classification results of the SOM does not provide good results when the SOM has not classified the samples correctly. The results also show that complete removal of the identified irrelevant input features produces worse class identification as shown in runs 1 and 4 in Table 5. This demonstrates the importance of identifying feature relevance based on class or at node level rather than a global SOM analysis and suggests the use of techniques that reduce the importance of identified irrelevant inputs rather than completely removing them.

A further experiment on the WSOM method with the WaveForm data that was used in the original WSOM paper has revealed that the method is able to group the dataset occasionally (i.e. at some random iteration) during multiple runs, but that other runs show a different set of weightings with a different solution. This provides further evidence to support the conclusion reached of multiple local minimums in the method’s cost function. The same SOM size of 26 × 14 as used by [8] was used for comparison. These results indicate that the WSOM method should be used with caution and that multiple runs may be required depending on the underlying data set in order to ensure that the optimal results are found. In practice, it may be difficult to know when these have been obtained making the use of this method problematic.

The quantization error between the weight values and their matched classified input samples shows more potential for identifying important features; however, these results show that this approach will only work for certain types of data. One of the limitations of the proposed method is its inability to correctly identify irrelevant inputs for a SOM with inappropriate topology size and having highly misdiagnosed classes (i.e. multiple classes mapped to a single node). This implies the requirement for an incremental system with the ability to automatically adjust the SOM’s topology size during training, to allow the spread of class samples across nodes for more accurate input relevance analysis. It is also interesting to note for some of the synthetic datasets that the quantization method correctly identifies the important features despite not being able to correctly classify the groupings in the data. This suggests the need for an additional layer that uses the relevance information from the feature selection method to prune or suppress the irrelevant features and guide the remapping of a self-organising system with the relevant features for a higher clustering performance to achieve a fully automated clustering process, and this will be the subject of future work.
